# A German e-learning-training in the context of early preventive intervention and child protection: preliminary findings of a pre-post evaluation

**DOI:** 10.1186/s13034-016-0113-8

**Published:** 2016-08-05

**Authors:** Kristina Bressem, Ute Ziegenhain, Claudia Doelitzsch, Alexandra Hofer, Tanja Besier, Joerg M. Fegert, Anne K. Kuenster

**Affiliations:** Department for Child and Adolescent Psychiatry/Psychotherapy, Ulm University Hospital, Ulm, Germany

**Keywords:** Child abuse, Child neglect, Child maltreatment, Prevention, e-learning, Child protection, Early preventive intervention

## Abstract

**Background:**

In recent years, a number of government-sponsored initiatives have been implemented in Germany that are focused on early preventive intervention in child protection. In response to the need for interdisciplinary training in this area, the internet-based e-learning program “Early Preventive Intervention and Child Protection” was developed for professionals in the child welfare and health care systems working with families with infants and toddlers. The program is currently undergoing evaluation for effectiveness and user satisfaction.

**Methods:**

In a pre-post design, users are requested to complete questionnaires that assess three measures of expertise: theoretical knowledge of relevant fields, the ability to correctly identify subtle signals of infant communication, and the ability to assess maternal sensitivity. This article presents the contents of the program and the pre-training results (N = 1.294 participants). Descriptive analyses as well as Pearson correlations and Bonferroni corrections of error were conducted using the statistical program SPSS v. 21.0.

**Results:**

The findings show that a wide range of professionals are making use of the program, and that their existing theoretical knowledge about early preventive intervention, as well as their ability to identify subtle signals of infant communication, is relatively good. However, their ability to assess maternal sensitivity, which is considered a crucial indicator for the risk of child abuse, was low.

**Conclusions:**

The outcome of the pre-training results indicates that professionals working in the area of child protection need to develop more capability in recognizing maternal sensitivity, in order to ensure early detection of families who are at risk and thus in need of support. Finally, the number of years of professional experience did not correlate with the scores on any of the three measures, which emphasizes the importance of providing interdisciplinary training in this area for all those working in child and family services, regardless of background.

## Introduction

### Early preventive intervention and child protection in Germany

Following some tragic cases of child abuse and neglect over the last decade in Germany that received intense media attention, policy-makers, researchers, and professionals who work with children and families became increasingly focused on the causes of child maltreatment and on possible strategies for its prevention. Early intervention is recognized as being especially desirable, as the problem of child abuse is most frequent and has the most detrimental consequences in infancy [1]. Children suffering from problematic early relational experiences with the primary caregiver are at higher risk for impaired cognitive development as well as for problems in emotional regulation, such as aggressive behavior, impulsiveness, and passivity [2]. A major factor is insensitive parenting behaviors that are controlling, hostile, or unresponsive [3–5].

To foster a preventive support system within the existing structures of health care and child welfare agencies, a number of political initiatives have been implemented in Germany at both the national and local levels. These initiatives were framed by a recently improved understanding of the significance of early preventive intervention and thus commonly called “Early Preventive Intervention”. In 2006, the Ministry for Family Affairs, Senior Citizens, Women and Youth initiated a program titled “Early Preventive Intervention for Parents and Children and Social Early Alert Systems” [6], which provided funding for pilot projects aimed at the prevention of child abuse and neglect. An evaluation of these pilot projects has been completed [7].

Experience with all these initiatives and projects has made it clear that early preventive intervention focused on child protection is a complex and demanding task, and one that cannot be fulfilled successfully through isolated initiatives or single disciplines [8]. Accordingly, there is a demand for cooperation between different professions in the areas of child welfare and health care. The German Child Protection Law, implemented on January 1st, 2012, created a national framework for this interdisciplinary approach, and marks a crucial step towards a systematic improvement in child protection [9]. The main focus of this law is the establishment of an interdisciplinary, comprehensive, high-quality, and easily accessible support system for all families that starts prenatally and extends through early childhood [10].

To achieve this challenging goal, it is clear that comprehensive interagency expertise is required, and that all professionals concerned with child and family care need to acquire a deeper understanding of preventive child protection including early signs of child abuse and neglect, and evidence-based support strategies. Successful prevention efforts rely on interdisciplinary knowledge and on competence at identifying known risk factors and existing warning signs at an early stage, in order to be able to protect children from further experience of violence. Interagency collaboration and networking are prerequisites, and educational programs are required that can provide systematic and interdisciplinary training for all impacted professionals.

## Background

### The e-learning program “Early Preventive Intervention and Child Protection”

The early parent–child relationship is a crucial focus in the area of child protection, because the fit between a child’s signals and the parent’s response crucially affects the development of social and cognitive functions [12]. The early detection of problematic relationships can help to identify parent–child dyads at risk for child maltreatment. Multiple international metaanalyses and reviews have shown that early intervention improves parenting competencies in general and reduces the probability of deviant parenting behavior [13–20], with attachment-based interventions that focus on improving the sensitivity of parental behavior during early infancy proving particularly effective [21]. Since knowledge about how to assess and improve the early parent–child relationship is relatively new to all disciplines, a central goal of a training program is to share this information with all professionals who work with families at risk.

Online training (e-learning) programs are ideal for providing training that is broad, systematic, and easily accessible. Users have complete flexibility with respect to when and where they access a course [22]; can individualize their learning by selecting content that is relevant to them; and can subsequently test their knowledge through interactive feedback [23]. Participants who are geographically dispersed can communicate with each other and can work in groups to solve tasks; a collaborative approach that accommodates the need for systematic education of different types of professionals involved in child protection [24].

“Early Prevention and Intervention in Child Protection” [11] is an innovative e-learning program that offers extra-vocational training for all professionals in the area of child protection, and thus meets the requirements for multidisciplinarity. The program was developed at Ulm University Hospital for Child and Adolescent Psychiatry/Psychotherapy, and was launched in June 2011. Its development and implementation were supported by the Ministry of Labour, Social, Families’, Women’s and Senior’s Affairs of the state of Baden-Württemberg in Germany. Since 2014, it has been sustainably financed through the UBS Optimus Foundation, and will continue to be funded until 2017 so that all interested participants can use it free of charge. By enabling individuals to access the training whenever, wherever, and at whatever pace they want, the program can reach high numbers of professionals in the child welfare and health care systems, thereby fostering a dissemination of important knowledge and practical know-how. It actively engages participants through the use of interactive media, such as showing videos of mothers interacting with their children and by presenting case studies that professionals can relate to their own work.

### Theoretical and practical content of the training program

The goal of the Early Prevention and Intervention in Child Protection e-learning program is to convey theoretical knowledge along with practical strategies on communication and interventions. The topics included in the e-learning program were selected on the basis of several action-research projects in the field of preventive child protection we conducted before (e.g. [8, 25–30]) and in discussion with an interdisciplinary advisory board including researchers as well as practitioners working in the field of child protection. The program comprises 90 training units, organized into the following categories:

1. Basic information: Interdisciplinary knowledge in the area of early intervention in child protection; e.g., the prevalence of child abuse and neglect, key stakeholders, basics of developmental psychology, risk factors and protective factors, applicable laws, and regulatory frameworks.
2. Manuals: Detailed explorations of specific topics from the basic information section, such as a parent with psychiatric problems, preterm birth, a child with physical or mental disabilities, attachment disorders, and regulation disorders.
3. Methods: Strategies for recognizing the signals that infants use to express their needs and emotions and for assessing a parent’s level of sensitivity to these signals, so that the quality of the parent–child interaction can be determined. This section includes a training tool for the evaluation of a child’s developmental status, and presents video examples to help train the user in communication skills with the parent.
4. Instruments for practical use: Strategies that can be employed in screening for risk factors, in evaluating a family’s need for professional support, and in locating services that are tailored to the family’s specific needs.
5. Glossary: Definitions of terms and concepts, in order to promote a common language and understanding across different disciplines.
6. Case vignettes: Presentation of 20 interactive vignettes that present relevant problems and questions from the perspective of diverse professions (e.g., social worker, psychologist, midwife, pediatrician, nurse). In addition to enabling an understanding of different perspectives and describing potential opportunities and pitfalls, these vignettes are designed to enhance the management and support of children facing a range of types and severities of maltreatment.

Furthermore, all users registered can communicate directly with each other on the platform.

As users work their way through the program, their progress is recorded graphically in a personal profile that displays their current status. Each of the 90 training units takes about 45 min to complete, and is followed by a multiple-choice test. Participants who achieve a minimum average of 70 % on all the tests receive a certificate indicating that they have successfully completed the course. Users can go through the program at the path they want, however, in order to get the certificate users have to finish all 90 units. In addition, those who are eligible for CME credits can claim 86 credit-hours, as approved by the regional medical association. The e-learning program contents a big variety of topics and methods which can be dealt with in every order the user choses. Thus, it is possible to use only parts of the e-learning program and pick the content which the user wants to work through.

In a pilot study of the e-learning program that evaluated 45 users [31], participants reported that they had gained significant knowledge and that they were using this knowledge to enhance aspects of their everyday practice. Specifically, they felt more secure in their assessments of the severity of cases; they were able to elicit better cooperation with other professionals and they were paying more attention to infants’ subtle signs of communication and to parental sensitivity.

### Evaluation of the e-learning program

The most valid outcome measure for evaluating the effect of the e-learning training program would be a change in the number of maltreated children in the impacted regions. However, this would require large-scale controlled studies, and would rely on unsupported assumptions about the accuracy of the data on the prevalence of child abuse and neglect in Germany. Accordingly, the program is instead being evaluated by conducting pre-post assessments of participants’ knowledge and understanding of intervention strategies in child protection. The findings will help to answer the following questions: Does participation in the e-learning program enhance professionals’ knowledge in the field? Do participants perceive the program as being helpful and effective? And is the program changing anything about how professionals decide on what interventions to implement, and in their confidence in these decisions?

The complete evaluation will be based on data provided by all participants who register for the e-learning program between November 2014 and December 2015. This report presents data from the pre-training questionnaire in order to learn something about the group of users interested in the training, to gather information on specific needs and potential gaps of knowledge and to figure out the need of specific training of observational skills in the field of preventive child protection for all professionals working with infants and toddlers. The final results of the pre-post comparison are expected to be available in March 2017, and will be published in a second paper.

## Methods

### Procedure

Users of the e-learning program were recruited through flyers that were mailed out to a range of professionals and institutions, such as midwife associations, youth centers, child protection agencies, pregnancy and early childhood counseling centers, church- and state-sponsored family counseling centers, nanny services, nursery schools, law firms, and many others. The geographical regions targeted were locations in Germany, Switzerland, and Austria. Respondents are assigned individual subject codes that are associated with their data, so that the information they provide is anonymized and is stored separately from their personal identifying information. The design of the evaluation was reviewed by the ethics committee of the Ulm University Hospital. The evaluation consists of pre- and post-training questionnaires and will answer the following questions:

Pre-Questionnaire:

- Who is taking part at the training?
- What are the needs and potential gaps of knowledge of the users?
- Do they need special training of observational skills?

Pre-post-comparison:

- Does participation in the e-learning program enhance professionals’ knowledge in the field?
- Is the program evaluated as being helpful and effective for professionals?
- Does the program change anything about how professionals decide on what interventions to implement, and in their confidence in these decisions?

Furthermore it will be asked what reasons are keeping some from completing the training. As part of the evaluation, data are collected on participants’ profession and place of residence, in order to determine if all targeted groups and regions are being reached.

### Questionnaires

An overview of the evaluation design is shown in Table 1. The pre-questionnaire appears automatically when a user enrolls, and must be completed in order to gain access to the course. It solicits information on the participant’s socio-demographics, current experience in the field of early preventive intervention and child protection, goals with respect to the use of the e-learning program, and the amount of time expected to be invested in it. The items for the questionnaire had been selected on the basis of the pilot-study mentioned above [31], which showed that the user learned while using the platform and stated, that they changed aspects of their everyday routine. Using a fictitious case we will be able to evaluate newly acquired observational skills. The evaluation of knowledge consists of the following:

**Table 1:** Design of the evaluation study

Type of questionnaire	Time point	Required for	Contents	Dates
Pre-training	After first log-in	Access to course material	Socio-demographic data Questions concerning goals, amount of time planned to invest in program, years of professional experience Questions concerning status of knowledge	November 2014 to December 2015
Post-training, Version A	After completion of all tests	Application for certificate	Knowledge questions Questions on practical use Questions on contentment with training	November 2014 to December 2016
Post-training, Version B	One year after completion of program Can be completed without having completed Version A	Resumed access to course material	Knowledge questions Questions on practical use Questions on contentment with training	November 2014 to December 2016
Non-completers	After 3 months without completion of a test or less than two logins	Not applicable	Questions about motivation and interest concerning the program	February 2015 to December 2016

- Theoretical knowledge, assessed through 24 questions to determine the user’s current state of understanding of the course material. Examples include: “What are intuitive parental competencies?”, “What is one of the problems in defining the term “child neglect?”, “How do unrealistic parental expectations affect a child’s development?”, and “What are the consequences to an infant when the parent’s style of interacting is potentially dangerous, e.g., due to borderline personality disorder?”
- Ability to recognize an infant’s subtle signs of communication, presented in a picture of an infant, lying on his back, with an averted gaze to the right, his hand rolled up to a fist and put to his mouth, while his left hand is holding a stuffed animal. Participants are asked to fulfill observational tasks by assigning the infant’s possible subtle signs of communication to different categories: (a) sign of approach, (b) sign of avoidance, (c) sign of self-regulation, (d) does not apply. The participants are shown a list of subtle signs, which should subsequently be identified in pictures of infants in different situations. Some examples of the portrayed infant’s possible subtle signs of communication include: “Putting hand in mouth/to head/to ear”, “making a fist”, and “averting gaze”.
- Ability to assess maternal sensitivity, using a case vignette that presents a short description of a family’s situation, including a video clip that shows a mother–child interaction. Based on the information provided, participants must rate the mother’s maternal sensitivity on 4 scales (sensitivity, emotional attunement, intrusive behavior, and non-responsive behavior), assess the family’s need for support, and indicate their confidence in this assessment.

The post-questionnaire is presented twice, differing only in the accompanying cover note. Version A automatically appears immediately after fulfillment of the course requirements, and must be completed in order for the participant to apply for the certificate, while Version B is accessed through a link that is emailed 12 months later. In both versions, participants are presented with the same knowledge questions, video clips of infant communication, and case vignette that were provided in the pre-questionnaire, in order to assess the extent of knowledge and skills gained. They are also asked whether they have made any practical use of the course information in their professional duties, and what their overall satisfaction with the course has been. After filling out the questionnaire Version B, participants gain resumed access to course material. Users who within 3 months of registration have not completed any of the multiple-choice tests or who have logged into the program less than twice are sent an email containing a link to a different questionnaire. They are asked to indicate, anonymously, if they have any further interest in the program; if no, what the reasons are for this, and if yes, what has kept them from making more use of it and what circumstances might induce them to do so. The goal is to better understand users’ motivations for completing the program: e.g., acquiring the certificate vs. acquiring knowledge for use in their daily work. Additionally, the responses of different professional groups are compared: for example, physicians and psychotherapists are able to apply their hours of training toward the CME requirements of their professional associations, whereas those in other fields cannot. The ability to acquire CME credits might be a motivation for completing the training. Strategies that might serve as an incentive to other professional groups are being considered and are planned to be implemented.

### Statistical analysis

The analysis of the data is being conducted using the statistical program SPSS v. 21.0. Descriptive analyses, Pearson correlations, Bonferroni corrections of error as well as ANOVAs and Scheffé-Tests are being carried out.

## Results

### Participants

1294 participants completed the pre-training questionnaire, 92.3 % were female and 7.7 % were male. Mean age was 37.7 years (SD ± 10.5; range 18–67). The majority had a baccalaureate degree, either general (60.4 %) or vocational (26.2 %). About half of the participants had children.

The e-learning program was directed at professionals in the fields of child, family, and healthcare services. Respondents were from diverse professional backgrounds, with the highest representations in the areas of social pedagogics (20.3 %), social work (12.3 %), pedagogics (11.6 %), kindergarten teachers (10.4 %) and psychology (10.3 %). Also represented were pediatricians, occupational therapists, pediatric nurses, midwives, child and youth psychotherapists, and family lawyers, at less than 10 % each. The majority of users (87.6 %) were actively working in their profession, with about half of them working full time. The mean number of years of experience in the field was 11.0 years (SD ± 9.5; range 0–40).

While 75.3 % of the participants indicated that work with families played an important role, only 26.0 % regarded early preventive intervention as relevant to their profession, only 21.6 % regarded child protection as relevant, and only 14.5 % regarded the field of family counsel as relevant.

## Measures

### Knowledge questions

Of the 24 knowledge questions, the mean number answered correctly was 14.1 (SD ± 2.98; range 1–22). Eleven of the questions were answered correctly by at least 70 % of users; another nine by 40–69 %; and four by fewer than 40 %. On average, the sum of all questions answered correctly was 58.6 %. The frequencies of correctly answered questions of knowledge are presented in Table 2.

**Table 2:** Frequencies of correctly answered questions of knowledge

No.	Question	%
20	Which statement is correct: deformities of newborns	88.3
13	Which answer is correct? Social-pedagogical family support is a service	85.7
3	What does the concept of intuitive competences describe?	83.6
22	Which statement about regulation problems of young children is correct?	83.3
16	What is rather not part of a client-oriented handling of professional communication?	79.9
17	Which statement is correct: when parents are at psychological risk…	78.6
7	What is one of the reasons that the category of child neglect is associated with definition problems?	78.0
1	What is correct in context of ''learning in the first year of life''?	76.4
12	Why is the role of child services in context of early preventive intervention characterized as tense/difficult?	74.7
5	How doe inadequate expectations of a child affect a child’s development?	72.1
14	Which statement is correct: from a judicial perspective, an “endangerment of a child’s well-being” can be assumed when…	71.0
6	What is the correct procedure for caregivers when a suspicion of a post traumatic syndrome exists?	61.3
8	Which statement is correct: A burn caused by maltreatment can be identified…	57.1
9	How is a risk factor described by the social and human sciences?	50.1
15	Which of the following statements is correct?	49.3
18	How do babies adapt to their parents’ style of interaction that is potentially dangerous (e.g. with parents having the Borderline syndrome)?	46.4
21	Which statement does not apply?	42.7
23	Is there an interface between early learning initiatives and early preventive intervention?	41.7
24	What is one of the reasons why the behavior of newborns is perceived as rebuffing?	41.4
4	Which of the following statements applies the least to newborns and toddlers?	40.9
10	For the assessment of an endangerment of a child’s well-being, multiple central questions can be formulated, which allow an assessment of the entire situation of the child. Which…	34.7
11	Which statement about the offers of early preventive intervention is correct?	34.5
2	Which statement is correct: unsecure ambivalent attachment….	32.9
19	How do psychotic mothers differ in their conscious experience from depressed mothers?	1.4

The number of correctly answered questions varied significantly between the different professions^1^ [F (6; 1125) = 12.42, p < .001]: Physicians answered significantly more questions correctly than kindergarten teachers, midwifes and (social) pedagogics and social workers. Kindergarten teachers answered significantly less questions correctly than occupational therapists, psychotherapists, psychologists and (social) pedagogics and social workers, whereas psychotherapists gained more points than (social) pedagogics and social workers (see Table 3).

**Table 3:** Number of correctly answered questions of knowledge split up for the different professional groups

Profession	N	M	SD	Min	Max
Physicians	64	15.84	2.53	9	20
Kindergarten Teachers	166	12.89	3.14	5	19
Occupational/physio-therapists	70	14.39	2.58	8	19
Midwifes	77	14.08	2.72	6	20
Psychotherapists	50	15.74	2.57	11	22
(Social) Pedagogics, social workers	572	14.08	2.82	1	21
Psychologists	133	14.53	2.97	5	22

### Identification of subtle infant communication signals

Concerning the picture that portrayed an infant displaying subtle communication, a mean of 4.92 (SD ± 1.75; range 0–8) subtle signs of communication were identified correctly. Seven of the eight signals were correctly identified by at least 56 % of participants (Table 4). On average, the sum of all correct answers lies at 61.5 %.

**Table 4:** Frequencies of correctly identified subtle infant communication signals

No.	Questions	%
4	Placing the hand into the mouth/to the head/to the ear	92.5
3	Holding on to an object	76.4
1	Looking away, aside, glance aversion sideways	64.8
7	Being awake	63.0
2	Overstretching	62.4
6	Rowing with arms	59.8
8	Rubbing eyes	56.8
5	Making a fist	16.3

Again the number of correctly identified subtle signs of communication varied significantly between the different professions [F (6; 1125) = 3.84, p < .001]: Psychologists answered significantly more questions correctly than kindergarten teachers and (social) pedagogics and social workers (see Table 5).

**Table 5:** Number of correctly identified subtle signs of infant communication split up for the different professional groups

Profession	N	M	SD	Min	Max
Physicians	64	4.83	1.72	1	8
Kindergarten teachers	166	4.61	1.66	0	8
Occupational/physio-therapists	70	5.16	1.71	1	8
Midwifes	77	5.21	1.55	1	8
Psychotherapists	50	5.18	1.78	1	8
(Social) Pedagogics, social workers	572	4.85	1.79	0	8
Psychologists	133	5.45	1.68	1	8

### Rating of parental sensitivity

Of the four ratings for maternal sensitivity, a mean of 0.97 (SD ± 0.86; range 0–4) were answered correctly. Between 10.9 and 37.0 % of participants answered the individual assessments of sensitivity correctly (Table 6). The overall average of all correct assessments was 24.4 %. The majority of participants (84.6 %) correctly assessed the family’s need for support, but only 58.5 % described themselves as “fairly sure” to “very sure” of their assessment, while 41.5 % were “fairly unsure” to “very unsure”.

**Table 6:** Frequencies of correctly assessed sensitivity

No.	Observational tasks	%
1	Maternal sensitivity	37.0
4	Non-responsiveness of the mother	25.6
2	Emotional attunement of the mother	24.0
3	Intrusive behavior of the mother	10.9

There were no differences in the competence of assessing maternal sensitivity between the professional groups (F (6; 1125) = 0.98, p = .440).

### Correlations between scores and between professional experience and scores

Pearson correlations were used to look for relationships between the number of correct answers given for each of the three measures. A slight correlation was found between scores on the theoretical knowledge questions and the observation of infants’ communication signals, in that the more questions were answered correctly, the more signals were identified correctly. No correlation was found between the ability to correctly assess maternal sensitivity with either of the other two measures. See Table 7.

**Table 7:** Pearson correlations between the number of correct answers on knowledge questions, correctly identified subtle infant communication signals, and correct assessment of maternal sensitivity

	Number of correctly observed infant signals	Number of correctly assessed ratings of maternal sensitivity
Number of correctly answered knowledge questions	.157*	.037
Number of correctly assessed ratings of maternal sensitivity		−.007

* p < .001 according to alpha-error correction by Bonferroni with three tests

Bonferroni corrections were used to determine the relationship between years of professional experience and the number of correct answers in each knowledge category. According to the three statistical tests conducted, there was a significant correlation between years of experience and the scores on the theoretical knowledge questions: the more experience the people have, the more they know. See Table 8.

**Table 8:** Pearson correlations between years of professional experience and the number of correctly answered knowledge questions, correctly observed subtle infant communication signals, and correctly assessed maternal sensitivity

	Number of correctly answered knowledge questions	Number of correctly observed infant signals	Number of correctly assessed ratings of maternal sensitivity
Years of professional experience	.125*	−.059	−.008

* p < .001 according to alpha-error correction by Bonferroni with three tests

## Discussion

The internet-based training program “Early Preventive Intervention and Child Protection” was implemented in Germany in response to the need for interdisciplinary training in this field. This paper presents pre-training data from the ongoing evaluation of the program, giving important information on how specialized professionals already are prior to the e-learning training and thus, pointing to the need of specialized training including skills training instead of providing only theoretical knowledge.

The e-learning program delivers relevant theoretical information; provides illustrations on topics such as parental mental illness, premature births, and children’s disabilities; employs a range of media methods; provides practical tools, such as ways to assess parent–child interactions and to communicate with parents; and includes systematic screening tools, a glossary of important terms relevant to the area of child protection, and a range of interactive case studies.

The pre-training results of 1.294 participants demonstrate a relatively solid state of existing knowledge, with an average of 14.1 correct answers out of 24 (about 60 %) on the theoretical knowledge questions, and an average of 5 correct answers out of 8 on the ability to correctly interpret the subtle language of infant communication. Some differences between the professions were found with physicians showing the highest number of correctly answered knowledge questions and psychologists with the best observational skills regarding the subtle communications signs of infants. Depending on the professions’ knowledge and observational skills, we expect differential benefits from our program. One contributing factor for the relatively good pre-training scores may be that the self-selected participants who chose to enroll in the program are those who have a high interest in further educating themselves in the field of child and family care. However, there are still gaps in knowledge and skills that could be filled by the e-learning program. The ability to assess maternal sensitivity, a crucial indicator for child development [4], was poor regardless the professional background of the participants, with a mean score of slightly less than 1 out of 4. Furthermore, while most participants correctly assessed the need for family support indicated in the case vignette, many were uncertain about their assessment. It seems that there is great potential for further training, especially concerning professionals’ skills.

The results found a correlation between scores on the questions of theoretical knowledge and on the ability to assess infants’ communication signals and between years of professional experience and the number of correctly answered knowledge questions. No correlation was seen between either of these scores and the ability to assess maternal sensitivity. This might be explained with the fact, that observational skills cannot be acquired theoretically but need practical training. Thus, our e-learning program includes many video clips, pictures and cases to train these important skills.

## Conclusions

Any conclusions at this stage are limited, as the evaluation is still in progress and no post-training data are yet available. Apart from this, a methodological limitation of the program evaluation is that a control group is not included. However, a strength is the large sample size, with the N of 1.294. The pre-data of the ongoing evaluation showed, that there is a need to improve providers’ understanding or assessment of maternal sensitivity. By making use of this e-learning training program to better educate themselves in the field of early preventive intervention, professionals working with children and their families should gain confidence in assessing the needs for support. The program described here is aimed at developing these assessment skills as well as improving the confidence needed to apply them. Since 2011, a total of 7.355 users have registered for the e-learning program; 873 have successfully completed it and earned the certificate, and 2.929 are currently enrolled. Once the currently ongoing evaluation is completed, the results should show if this program is indeed enabling professionals to significantly improve their everyday routines in ways that will allow them to help families at risk in a more accurate and suitable manner.

## Footnotes

1 Only professions with at least 20 participants were included in the analyses.
